# Common and specific downstream signaling targets controlled by Tlr2 and Tlr5 innate immune signaling in zebrafish

**DOI:** 10.1186/s12864-015-1740-9

**Published:** 2015-07-25

**Authors:** Shuxin Yang, Rubén Marín-Juez, Annemarie H. Meijer, Herman P. Spaink

**Affiliations:** Institute of Biology, Leiden University, PO Box 9502, 2300 RA Leiden, The Netherlands; ZF-screens BV, J. H. Oortweg 19, 2333 CH Leiden, The Netherlands; Present address: Department of Developmental Genetics, Max Planck Institute for Heart and Lung Research, Ludwigstrasse 43, 61231 Bad Nauheim, Germany

**Keywords:** TLR signaling, Zebrafish, TLR2, TLR5, Flagellin, Pam3CSK4, Innate immunity

## Abstract

**Background:**

Although the responses to many pathogen associated molecular patterns (PAMPs) in cell cultures and extracted organs are well characterized, there is little known of transcriptome responses to PAMPs in whole organisms. To characterize this in detail, we have performed RNAseq analysis of responses of zebrafish embryos to injection of PAMPs in the caudal vein at one hour after exposure. We have compared two ligands that in mammals have been shown to specifically activate the TLR2 and TLR5 receptors: Pam3CSK4 and flagellin, respectively.

**Results:**

We identified a group of 80 common genes that respond with high stringency selection to stimulations with both PAMPs, which included several well-known immune marker genes such as *il1b* and *tnfa*. Surprisingly, we also identified sets of 48 and 42 genes that specifically respond to either Pam3CSK4 or flagellin, respectively, after a comparative filtering approach. Remarkably, in the Pam3CSK4 specific set, there was a set of transcription factors with more than 2 fold-change, as confirmed by qPCR analyses, including *cebpb*, *fosb*, *nr4a1* and *egr3*. We also showed that the regulation of the Pam3CSK4 and flagellin specifically responding sets is inhibited by knockdown of *tlr2* or *tlr5*, respectively.

**Conclusions:**

Our studies show that Pam3CSK4 and flagellin can stimulate the Tlr2 and Tlr5 signaling pathways leading to common and specific responses in the zebrafish embryo system.

**Electronic supplementary material:**

The online version of this article (doi:10.1186/s12864-015-1740-9) contains supplementary material, which is available to authorized users.

## Background

The innate immune system is referred to as the first line in host defense against invading pathogens [1, 2]. Its highly developed ability to recognize microbial patterns and host-derived danger signals relies on so-called pattern recognition receptors (PRRs), especially on the Toll-like receptors (TLRs) [3–5]. In humans, the TLR family is composed of 10 members, which are located at the cell surface with the exception of TLR3, 7, 8 and 9, which are localized on intracellular endosomal membranes [6–8]. The TLRs are involved in the recognition of a wide variety of ligands, including pathogen associated molecular patterns (PAMPs), such as bacterial cell wall components and viral RNA, as well as damage associated molecular patterns (DAMPs) [9, 10]. This leads to subsequent intracellular signal transduction, triggering the production of inflammatory cytokines and chemokines, but it can also lead to anti-inflammatory responses as has been recently shown for TLR10 acting as a heterodimer with TLR2 [11, 12].

The recognition of PAMPs and DAMPs by different TLRs is directed by structurally conserved leucine-rich repeats (LRR) motifs of the TLRs ectodomains (ECDs) [13, 14]. Cell-surface TLRs can mediate binding to PAMPs by homodimerizing, like TLR4 and TLR5 that recognize lipopolysaccharide (LPS) [15] and flagellin [16], respectively. In contrast, other TLRs form heterodimers like TLR2 that associates with TLR1, TLR6 or TLR10, in conjunction recognizing lipopeptides and lipoproteins [12, 17, 18]. The diversity of TLR2 dimer combinations is thought to be responsible for its extensive recognition ability, ranging from the diverse components of various pathogens to the host heat-shock protein 70 [10]. TLR2 plays an important role in resistance to the infection induced by *Mycobacterium tuberculosis* (*Mtb*) [19, 20]. For example, McBride et al. demonstrated that TLR2 knockout mice show enhanced cell infiltration and inflammation in lungs upon low dose chronic infection with *Mtb*, and fail to stably control the bacterial burden [21]. A series of components of *Mtb* can trigger the TLR2 signaling pathway upon infection, such as triacylated lipoprotein LprG, LpqH and PhoS1 [22, 23], and glycolipid lipoarabinomannan [24]. Pam3CysSerLys4 (Pam3CSK4) is a synthetic triacylated lipopeptide that mimics the triacylated lipoprotein of mycobacteria and classical gram-positive bacteria, which can be recognized by TLR2/TLR1 heterodimers and induce the production and release of pro- and anti-inflammatory cytokines (IL-6, IL-12, TNF-α and IL-10), chemokines (IL-8) and interferon (IFN-γ) [25–28]. Most of these studies on the responses of the TLR2 signaling pathway have been performed in cell culture systems. A notable exception is the reported transcriptome response of mouse mononuclear phagocytes recruited to lungs challenged with Pam3CSK4 as measured by micro-arrays [29]. As another example 8 day old mice were treated with Pam3CSK4 and analyzed for the expression of several inflammatory genes using qPCR [30].

The zebrafish embryonic model has much potential to study the ligand specificity of TLRs at the organism level [29]. Importantly, zebrafish offers the possibility to study the innate immune system separated from the adaptive immune system in their embryonic and larval stages (up to 3-4 weeks post fertilization) [31–33]. To date, a number of TLR signaling pathway mediators have been identified and studied in zebrafish such as the adaptor proteins Myd88, Tirap (Mal), Trif and Sarm1 [34–38], and the downstream signaling intermediates Irak and Traf6 [39, 40].

In work previously published by our group, we demonstrated that the function and regulation of the zebrafish homologs of human TLR5, *tlr5a* and *tlr5b*, are conserved with their mammalian counterparts. Both *tlr5a* and *tlr5b* are strongly up-regulated in response to *Salmonella typhimurium* infection [41]. Furthermore, in the same study it was shown that knockdown of these two genes prevented or weakened the activation of genes for several inflammatory mediators like *mmp9*, *cxcl-C1c*, *il1b* and *il8* upon flagellin stimulation [41].

In this study, we aimed to study TLR2 function, in comparison with TLR5, in zebrafish using transcriptome analysis. Injection into the blood stream of the *tlr2/tlr1* ligand Pam3CSK4, was followed by transcriptome profiling to characterize key genes involved in the early response to this PAMPs. In addition, by comparing the transcriptome response towards treatment with flagellin, we were able to discriminate non-specific immune responsive genes from a set of genes which are regulated by *tlr2* but not by *tlr5*.

## Methods

### Zebrafish husbandry

Wild-type zebrafish of the AB/TL strain were handled in compliance with the local animal welfare regulations and maintained according to standard protocols (zfin. org). Embryos were raised in egg water (60 g/ml Instant Ocean sea salts) at 28.5 °C. For the duration of bacterial injections, embryos were kept under anesthesia in egg water containing 0.02 % buffered 3-aminobenzoic acid ethyl ester (Tricaine). The breeding of adult fish was approved by the local animal welfare committee (DEC) of the University of Leiden. All protocols adhered to the international guidelines specified by the EU Animal Protection Directive 2010/63/EU.

### Morpholino injections

Morpholino oligonucleotides (Gene Tools) were diluted to desired concentrations in 1× Danieu’s buffer (58 mM NaCl, 0.7 mM KCl, 0.4 mM MgSO_4_, 0.6 mM Ca (NO_3_)_2_, 5.0 mM HEPES (pH 7.6)) containing 1× phenol red (Sigma-Aldrich). For knockdown experiments, *tlr2* ATG-morpholino (*tlr2* mo, Additional file 1: Table S1) was injected with the optimal concentration at 0.5 mM and 1 nl volume per embryo at 0 ~ 2 cell stage*. tlr5* translation was blocked via injecting 1 nl of the *tlr5a* and *tlr5b* ATG-morpholinos at a dose of 0.1 mM and 0.5 mM at 0 ~ 2 cell stage, as previously published by *Stockhammer* and coworkers [41]. Control embryos were injected with the standard control morpholino (Sc mo, Additional file 1: Table S1).

### Ligands injection

For the ligands injection assay, purified Pam3CSK4 (InvivoGen) and flagellin from *S. typhimurium* (Flagellin FliC VacciGrade™, Invitrogen) were respectively diluted to 1 mg/ml and 100 μg/ml in sterile water. For their administration, 1 nl of the ligands was injected into the blood stream at 27 hpf, and sterile water was used as control. Injections were perormed using a FemtoJet microinjector (Eppendorf) and a micromanipulator with pulled microcapillary pipettes.

### RNA isolation, cDNA synthesis and qPCR

RNA was extracted using TRIzol Reagent (Life Technologies) and purified by column according to the manufacturer’s instructions of RNeasy MinElute Cleanup Kit (Qiagen). The concentration and quality of RNA were detected by NANODROP 2000/2000c (Thermo Scientific). 1 μg cDNA synthesis reactions and qPCR were performed as described in the manufacturer’s instructions (iScript™ cDNA Synthesis Kit and iQ™ SYBR® Green Supermix, BioRad) and normalized against the expression of *ppial* as a housekeeping gene [42]. PCR analysis was performed using the following protocol: 95 °C 3 min, 40 cycles real time of 95 °C 15 s and 60 °C 45 s, and final melting curve of 81 cycles from 95 °C 1 min to 55 °C 10 s. Results were analyzed using the ΔΔCt method. Primer sequences used can be found in Additional file 1: Table S1.

### Deep sequencing and data analyzing

Triplicates of 10 ~ 20 embryos of AB/TL or *tlr* morphants from three injection conditions, Pam3CSK4, flagellin or water injection, were homogenized in 500 μl of Trizol reagent (Qiagen). Total RNA was extracted and column-purified according to the manufacturer’s instructions of the RNeasy MinElute Cleanup Kit (Qiagen). The subsequent sample preparation and Illumina RNA sequencing were as previously described [43]. RNA samples were treated with DNaseI (Life Technologies) to remove residual genomic DNA. RNA integrity was analyzed by Lab-on-a-chip analysis (Agilent, Amstelveen, The Netherlands). A total of 2 μg of RNA was used to make RNAseq libraries using the Illumina TruSeq RNA Sample Preparation Kit v2 (Illumina, Inc., San Diego, CA, USA). The manufacturer’s instructions were followed with the exception of two modifications. In the adapter ligation step, 1 μl, instead of 2.5 μl, adaptor was used. In the library size-selection step, the library fragments were isolated with a double Ampure XP purification with a 0.7× beads to library ratio (Beckman Coulter, Woerden, The Netherlands). The resulting mRNAseq library was sequenced using an Illumina HiSeq2500 Instrument (Illumina, Inc.) according to the manufacturer’s instructions with a read length of 2 × 50 nucleotides. Image analysis and base-calling were done using the Illumina HCS version 2.0.12. The raw data has been submitted to the GEO database (accession number GSE64570). The total number of reads for each sample is summarized in Additional file 2: Table S4 and quality control was according to the sequencing company guidelines (ZF-sceens.com). The data was analyzed using the GeneTiles software (http://www.genetiles.com) [44] using a cut-off setting of 2 fold-change and a *p*-value <0.01. In brief, Genetiles used fastq files as input for the program Bowtie2 [44] to align the reads to the zebrafish genome (obtained from Ensembl version Zv9). Subsequently, the programs SAMtools [45], DESeq and DEXSeq [46, 47] are used for data processing. The complete data processing pipeline for Genetiles, including the used parameters, is available for download at www.genetiles.com and can also be found in Veneman et al [44]. Using these settings we have mapped the numbers of reads as shown in Additional file 2: Table S4. The triplicate data sets of Pam3CSK4, flagellin and control treatments were mapped to 27104, 26583, and 26409 ENSEMBL genes, respectively. The difference between the mapped reads of the individual samples compared with the mapped reads of triplicate samples was always lower than 12 % (Additional file 2: Table S4). GO analysis was performed using the software package DAVID available at http://david.abcc.ncifcrf.gov/home.jsp [48].

## Results

### The immune response of zebrafish embryos to injection of PAMPs in the caudal vein

**Fig. 1:**
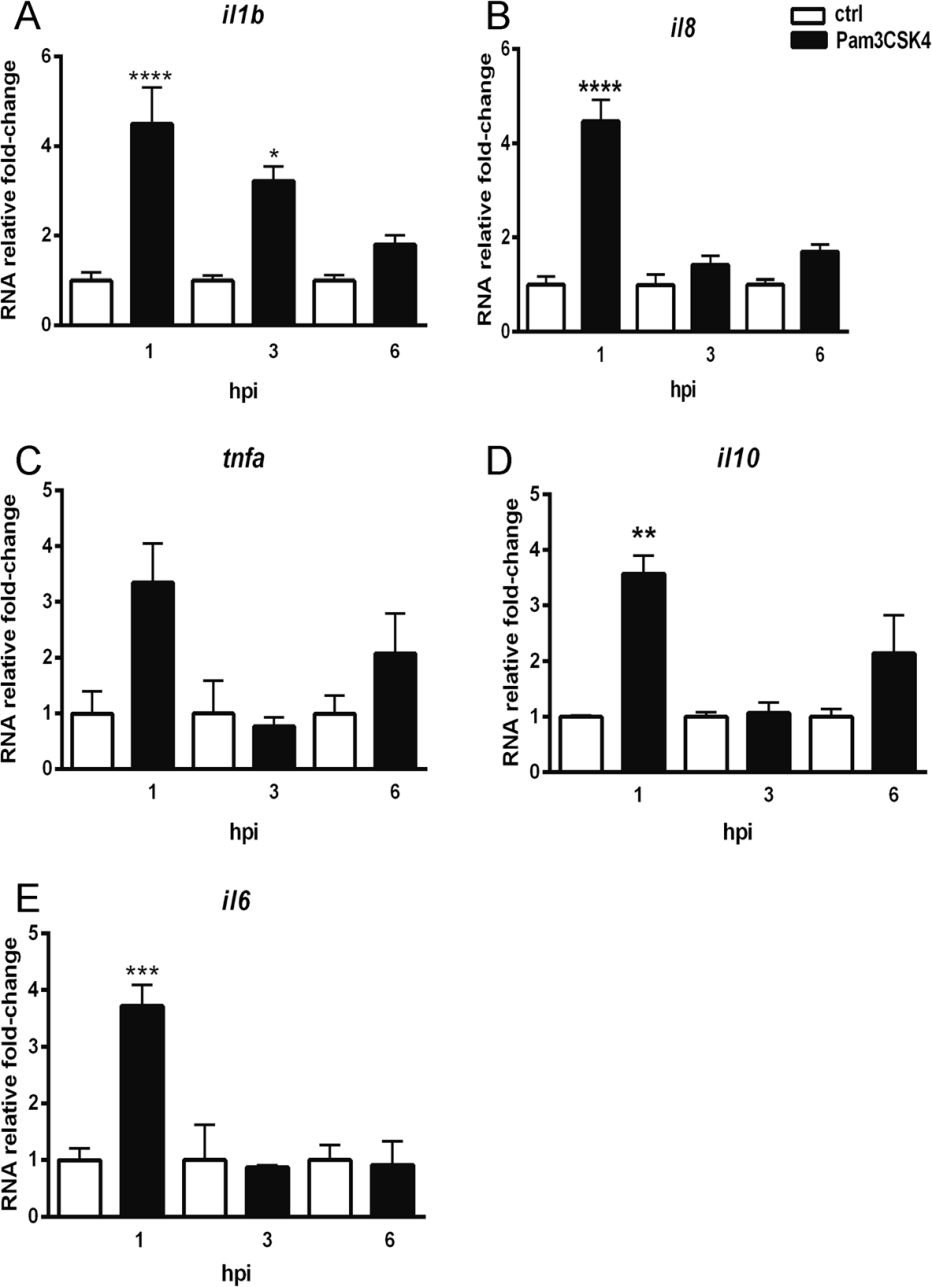
Immune genes expression at different time points upon Pam3CSK4 stimulation. Embryos were injected at 27 hpf with 1 ng Pam3CSK4 and expression levels of *il1b* (**a**), *il8* (**b**), *tnfa* (**c**), *il10* (**d**) and *il6* (**e**) were determined at 1, 3 and 6 hpi by qPCR. Data (mean ± SEM) are combined from at least three biological replicates (*n* = 15 embryos per group) and expressed relative to their corresponding mock injection (water) control, which is set at 1. Statistical significance of differences between mock and Pam3CSK4 groups was determined by ANOVA analysis and Tukey’s multiple comparisons test, *p < 0.05, **p < 0.01, ***p < 0.001

In a recent study by Stockhammer et al, it was shown that flagellin injected into the caudal vein at 27 h post fertilization (hpf) induced several immune response marker genes as measured by qPCR [41]. To further characterize the response to another well characterized PAMP, we injected Pam3CSK4 using the same method. The expression levels of cytokine genes *il1b*, *tnfa* and *il6*, the chemokine gene *il8,* and anti-inflammatory gene *il10,* were measured by qPCR at 1, 3 and 6 h post injection (hpi) respectively (Fig. 1). The results show that there was a significant up-regulation of these genes upon Pam3CSK4 stimulation. For all these marker genes the induction was transient and followed by a gradual decrease over time. The *il1b* gene was the only marker of which up-regulation was observed at 1 and 3 hpi, with a significantly higher expression than the control group (Fig. 1a). For the *tnfa*, *il6, il8* and *il10 genes* there was a more obvious decrease of induction over time (Fig. 1b–e). These results show that Pam3CSK4 induces similar responses in zebrafish as in mammalian cells [25–28] suggesting that this response is also mediated via the *tlr2* signaling pathway.

### The function of Tlr5 and Tlr2 in the immune response towards flagellin and Pam3CSK4

**Fig. 2:**
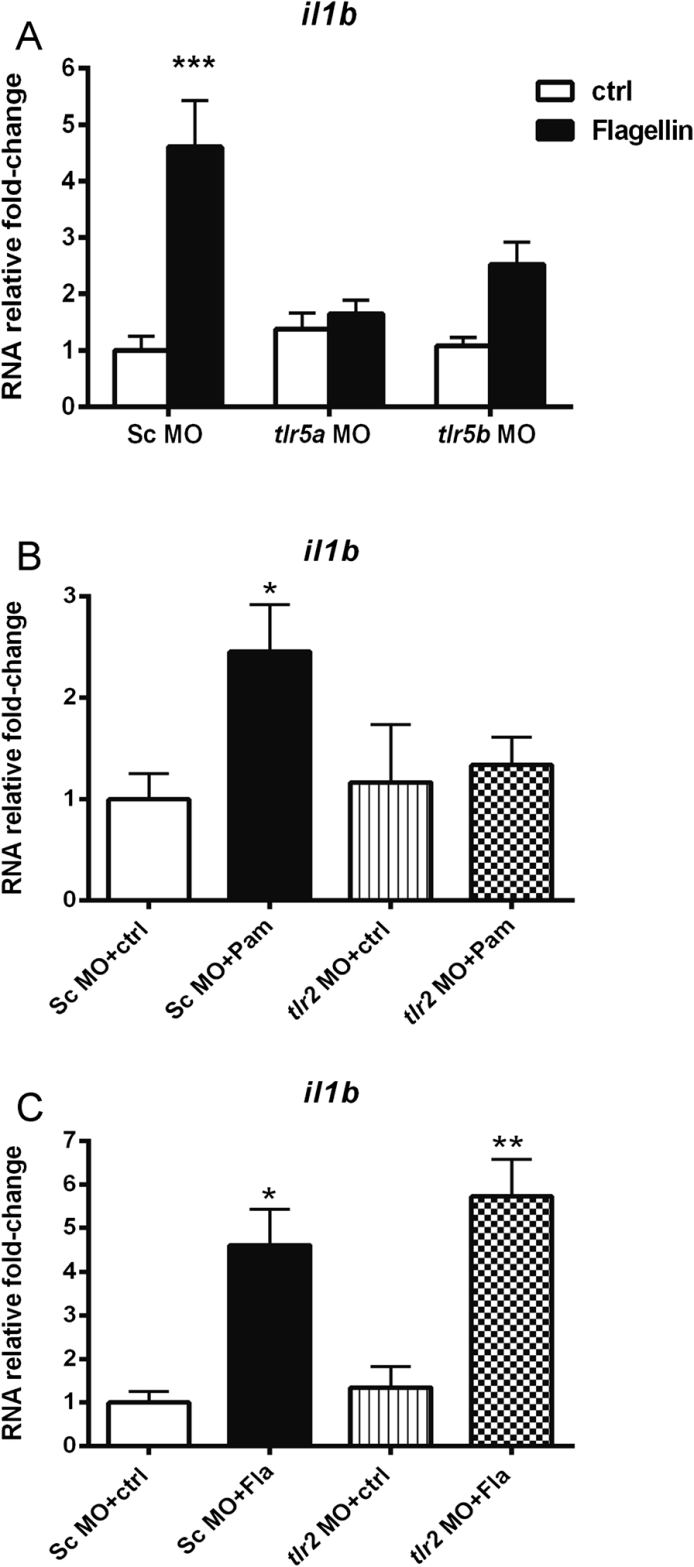
*il1b* expression in *tlr2* and *tlr5* morphants following PAMPs stimulation. Embryos were injected at the 1-2 cells stage with standard control (Sc), *tlr2, tlr5a or tlr5b* morpholino (MO) and subsequently injected with Pam3CSK4 at 27 hpf, flagellin or water as a mock control. Expression of *il1b* was determined by qPCR at 1 hpi. **a** *tlr5a* and *tlr5b* knockdown effect on *il1b* RNA expression in response to flagellin. **b** C *tlr2* knockdown effect on *il1b* RNA expression in response to Pam3CSK4 (**b**) or flagellin (**c**). Data (mean ± SEM) are combined from at least three biological replicates (*n* = 10 embryos per group) and expressed relative to their corresponding water control, which is set at 1. Statistical significance was determined by ANOVA analysis and Tukey’s multiple comparisons test, *p < 0.05, **p < 0.01, ***p < 0.001; Pam, Pam3CSK4 injecton; Fla, flagellin injection

In order to study the function of *tlr2* and *tlr5* in the above described responses to Pam3CSK4 and flagellin we used morpholinos to knockdown these genes. There are two orthologous genes of human *tlr5* in zebrafish, *tlr5a* and *tlr5b* and previous studies in our group showed that they are required for activation of host defense genes upon flagellin stimulation. This was shown by simultaneous co-knockdown of *tlr5a and tlr5b* by morpholinos [41]. In this study, *tlr5a* and *tlr5b* morpholinos were injected separately and, subsequently the morphants were stimulated with flagellin at 27 hpf. Embryos treated with standard control morpholino were used as a control [41]. The expression of *il1b* was measured at 1hpi by qPCR. Our results revealed that abrogation of both *tlr5a* and *tlr5b* effectively prevented the *il1b* up-regulation observed in control embryos upon flagellin stimulation (Fig. 2a).

The function of *tlr2* in recognition of Pam3CSK4 was tested in the same manner. Our results showed that *tlr2* morphants did not exhibit up-regulation of *il1b* expression upon Pam3CSK4 stimulation, while the control morphants did (Fig. 2b). In contrast, *tlr2* morphants stimulated with flagellin, still showed a significant induction of *il1b* expression (Fig. 2c).

### Identification of a common response gene set for Pam3CSK4 and flagellin stimulation

Since comparisons of the transcriptome response to PAMPs that activate different TLRs has not been described before in a whole organism we decided to perform RNA deep sequencing (RNAseq) of embryos treated with flagellin and Pam3CSK4 at 1 hpi.

**Fig. 3:**
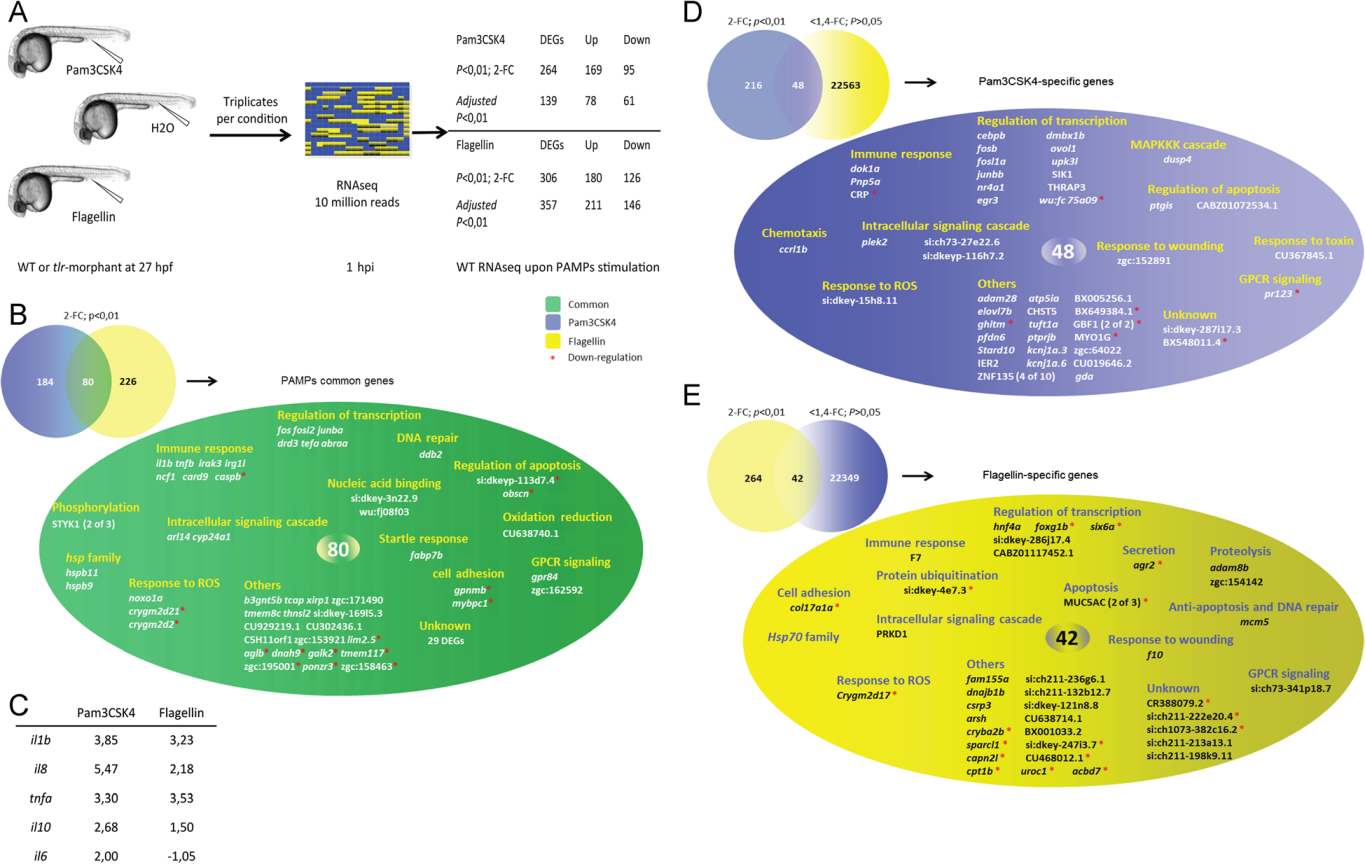
RNAseq experimental setup and comparison of gene sets responsive to Pam3CSK4 or flagellin stimulation. **a**, setup of the RNAseq experiment. Zebrafish embryos received a 1 nl injection of 1 mg/ml Pam3CSK4 and 100 μg/ml flagellin respectively into the caudal vein at 27hpf. Control embryos were injected with water. Samples for RNAseq were taken at 1hpi. The numbers of differentially expressed genes were assessed by two criteria: 1) *p* < 0.01, 2 fold-change or 2) adjusted *p*-value <0.01, without FC cut-off. **b** Venn diagram showing the overlap between DEGs from Pam3CSK4 and flagellin stimulations and their GO terms annotation. **c** Fold-change values of inflammatory genes in RNAseq. **d** Filtering of 264 DEGs from Pam3CSK4 stimulation (*p* < 0.01, 2 fold-change) by the flagellin non-specific set (22611 genes, *p* > 0.05; <1.4-fold change) results in 48 Pam3CSK4 specific genes, which are grouped according to their GO terms annotation. **e** Filtering of 306 DEGs from flagellin stimulation (*p* < 0.01, 2 fold-change) by the Pam3CSK4 non-specific set (22391 genes, *p* > 0.05; <1.4-fold change), results in 42 flagellin specific genes, which are grouped according to their GO terms annotation. DEGs, differentially expressed genes; FC, fold-change

Embryos injected with sterile water were used as control and RNA was isolated from a pool of at least fifteen embryos per condition. Triplicates of biological samples were analyzed with Illumina RNAseq and at least 7.2 million mapped reads were obtained for each library (Fig. 3a, Additional file 2: Table S4). Although such reads numbers are insufficient to detect changes in very lowly expressed genes (see Veneman et al, 2014 [49]) approximately 10 million total reads is currently a good cost efficient number that matches the sensitivity of microarrays [50]. The results of the transcriptional responses are summarized in Fig. 3a and Additional file 3: Figure S1. The results show that at any given *p*-value (or false discovery rate-adjusted *p*-value), flagellin leads to a higher number of differentially expressed genes (DEGs) than Pam3CSK4. To further analyze the data, we arbitrarily used a threshold of 2-fold change and *p-*value <0.01. This p-value corresponds to FDR adjusted p-values ranging from 0.23 to 0.35 in the different experiments. These selection criteria are not very stringent so as to prevent loosing genes that are very lowly expressed and therefore with the used sequencing depth will have obtained only low numbers of reads. The entire list of responses without any selection criteria is given in Additional file 4: Table S5 and Additional file 5: Table S6. Applying these settings we obtained 264 DEGs from the Pam3CSK4 stimulated group, composed of 169 up- and 95 down-regulated genes, and 306 DEGs from the flagellin injected group, composed of 180 up- and 126 down-regulated genes (Fig. 3a). Therefore, with both treatments there are more genes up-regulated than down-regulated. In the list of top induced and repressed genes there is a lack of any annotation in the data bases (Additional file 4: Table S5 and Additional file 5: Table S6). We compared these two groups of genes and found an overlap set of only 80 genes that include many immune marker genes, such as *il1b*, *tnfb*, *irak3* and *irg1l*, and transcription factors, like *fos*, *fosl2* and *junba*, as shown by the gene ontology terms (GO terms) annotation in Fig. 3b and Additional file 6: Table S3 .

The induction of *il1b*, *tnfa* and *il8* by Pam3CSK4 shown using qPCR (Fig. 1) was confirmed by the RNAseq data (Fig. 3c), but in the case of *il6* and *il10* there was no induction with flagellin*.*

### Identification of gene sets that are specifically regulated by Pam3CSK4 and flagellin

This study unexpectedly revealed that there is a relatively large group of genes that are only induced or repressed by either Pam3CSK4 or flagellin (Fig. 3b). To further test whether this specifically induced group is completely unaffected by the other PAMP treatment we used a rigorous filtering approach as shown in Fig. 3d and e. For this approach, the DEGs from the Pam3CSK4 (264 genes) and flagellin (306 genes) stimulation groups were compared with the group of genes that were not affected by flagellin (22611 genes) and Pam3CSK4 (22391 genes), respectively, with a cut-off setting at <1.4-fold change and *p* > 0.05. By taking the overlap of these sets we thereby exclude genes that were inducible by the other ligand even at very low stringency. This resulted in a set of 48 genes (Fig. 3d) for which the response is specific to Pam3CSK4 and a set of 42 genes (Fig. 3e) for which the response is specific to flagellin. GO analysis indicated that these two groups contain different categories (Fig. 3d, e). Most notably, genes with high fold-change (>2) from the Pam3CSK4 specific group include many transcription factors involved in the TLR signaling pathway, such as *junbb*, *cebpb*, *fosb*, *fosl1a*, *egr3* and *nr4a1*. In the flagellin specific group of genes this is not the case and an obvious enriched category could not be identified.

**Fig. 4:**
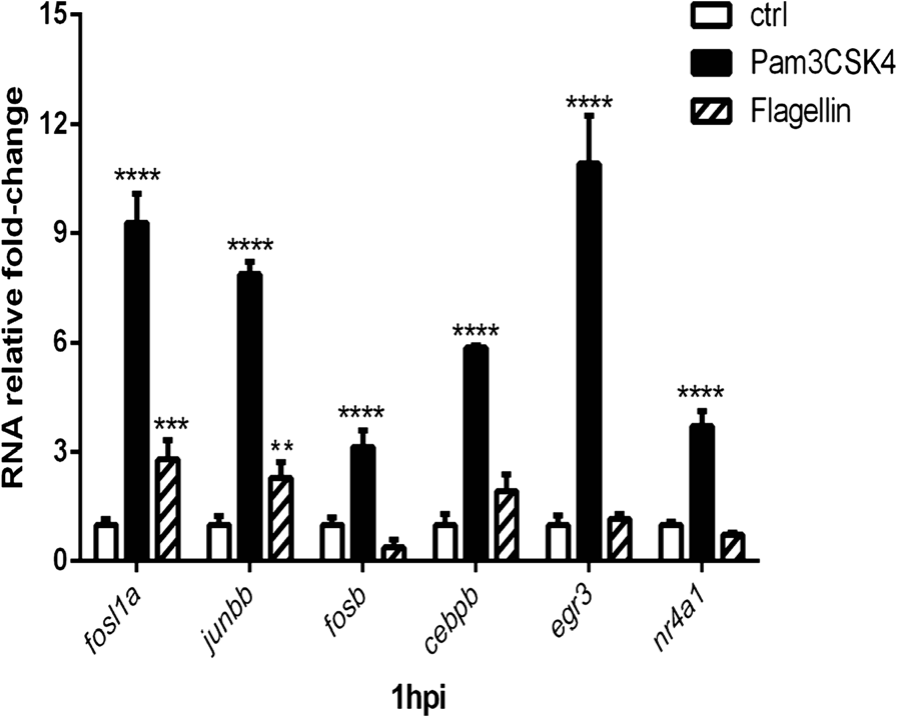
qPCR analysis of transcription factors genes responsive to PAMPs stimulation. Expression levels of *fosl1a, fosb, junbb, cebpeb, egr3* and *nr4a1* following Pam3CSK4 and flagellin stimulation are determined by qPCR. Data (mean ± SD) are combined from at least tree biological replicates (*n* = 15 embryos per group) and expressed relative to their corresponding water control, which is set at 1. Statistical significance was determined by two-way ANOVA analysis and Tukey’s multiple comparisons test, *p < 0.05, **p < 0.01, ***p < 0.001, ****p < 0.0001

To confirm the result of deep sequencing, qPCR was performed to verify the responses of genes from the Pam3CSK4 specific gene set, namely *junbb, cebpb, fosb, fosl1a, egr3* and *nr4a1* (Fig. 4). As expected, the expression level of all these transcription factors confirmed the deep sequencing result. Moreover, these genes did not exhibit an apparent differential expression in zebrafish upon flagellin stimulation except *fosl1a* and *junbb* (Fig. 4). Even though the expression of these both genes showed significant induction upon flagellin stimulation, the induction level was still far lower than that upon Pam3CSK4 stimulation.

### Function of the tlr2 and tlr5a genes in the transcriptome responses to Pam3CSK4 and flagellin

**Fig. 5:**
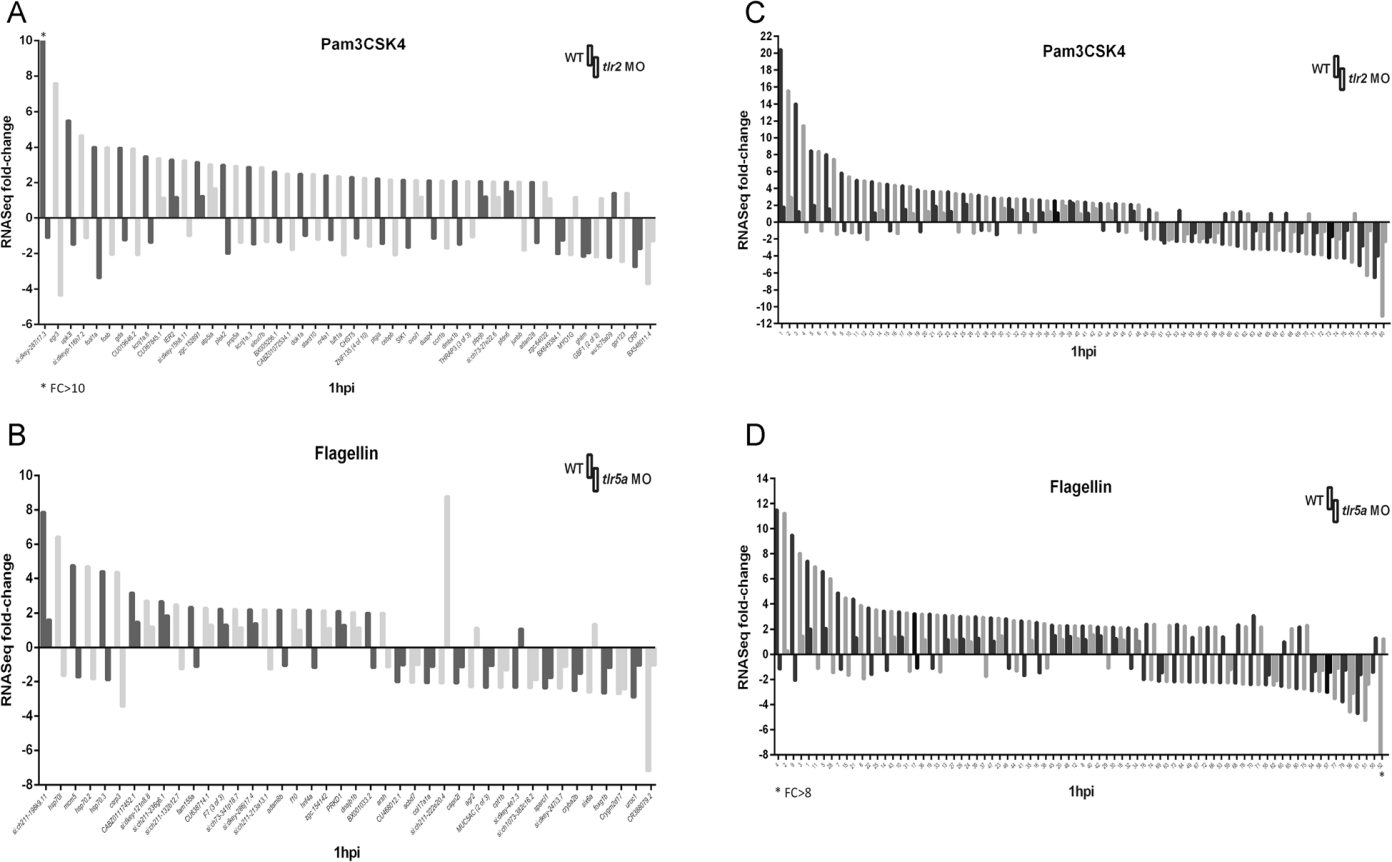
Effects on *tlr2* and *tlr5* knockdown on the expression of Pam3CSK4 and Flagellin responsive genes **a**, based on RNAseq data, all the 48 Pam3CSK4 specific fold-changes are inhibited or down-regulated in *tlr2* morphants (*tlr2* MO) upon this PAMP stimulation. **b**, based on RNAseq data, all the 42 flagellin specific fold-changes are inhibited or down-regulated in *tlr5a* morphants (*tlr5a* MO) upon this PAMP stimulation. **c**, **d**, all the 80 common fold-changes are inhibited or down-regulated in *tlr*2 and *tlr*5a morphants. FC, fold-change. Panel **a**, **b**, **c**, **d**: for the quantitative data and accession numbers of the shown genes (or numbers) we refer to Additional file 7: Table S2 and Additional file 6: Table S3

To confirm that the transcriptome responses upon PAMPs stimulation described above (Figs. 3b and 4) are *tlr2* or *tlr5* specific, we performed RNAseq analyses of the Pam3CSK4 and flagellin responses under *tlr2* and *tlr5a* knockdown conditions, again using biological triplicates of each group. Setting a threshold of 2 fold-change and *p* < 0,01, we found that the 80 common DEGs responsive to both Pam3CSK4 and flagellin were reduced by *tlr2* and *tlr5a* knockdown (Fig. 5c, d and Additional file 6: Table S3). Furthermore, all the 48 genes (40-up and 8-down-regulated) from the Pam3CSK4 specific group showed no longer a differential expression or an anti-correlated expression after *tlr2* knockdown and, similarly all the 42 genes (24-up and 18-dow-regulated) from the flagellin specific group showed no longer a differential expression or an anti-correlated expression upon *tlr5a* abrogation (Fig. 5a, b and Additional file 7: Table S2). Overall, these results confirm the specificity of both morpholinos and show that zebrafish *tlr2* and *tlr5a* are key mediators of the transcriptomic response triggered by injection of Pam3CSK4 and flagellin respectively.

## Discussion and Conclusion

The signaling pathways underlying recognition of PAMPs have been studied intensively and this has led to a broad understanding of key regulators of innate immunity based on studies of cell cultures and the use of knockout rodent mutants. The new possibilities for analysis of transcriptomes using RNA deep sequencing make it highly attractive to analyze the responses of an entire test animal model at the system biology level. In this manuscript we have chosen the zebrafish embryo model for such an approach and have included functional analysis of Tlr5 and Tlr2 in the response towards two well-known PAMPs, flagellin and Pam3CSK4.

The results show that there is a relatively limited overlap between the transcriptome responses towards flagellin and Pam3CSK4 (Fig. 3b). The overlap includes well known downstream immune mediators that were previously shown to be induced by flagellin [41] such as *il1b*, *tnfa*, *irak3, mmp9, cxcl-c1c* and *il8*. In contrast, *il6* and *il10*, that are associated with an anti-inflammatory response, were induced much stronger by Pam3CSK4 than by flagellin. A relatively much larger group of genes showed a differential response to flagellin or Pam3CSK4, including a group of genes of which the transcription is specific for activation by one of the two treatments (Fig. 4).

GO terms analysis of the genes specifically regulated by these two PAMPs show that there is an enriched category of transcription factors in the Pam3CSK4-specific group, of which most of genes are up-regulated and only one is down-regulated. Additionally, a less enriched category of immune response genes is found in this group as well, which include a down-regulated CRP (C-reactive protein). For the flagellin-specific group of genes, there are only five genes under the GO-term regulation of transcription of which two are down-regulated. Many of the genes specifically induced by Pam3CSK4 have also been shown to be strongly regulated by infection in the zebrafish embryo system [51] and therefore we would like to further study this group of genes in more detail in future research.

For functional analysis of the transcriptome response towards flagellin and Pam3CSK4 we used morpholinos that were selected on basis of their blocking effect on downstream signaling using qPCR and subsequently confirmed by RNAseq analysis. Surprisingly for *tlr5* we found that a morpholino against each of the two copies of this gene, *tlr5a* and *tlr5b* had an effect on downstream signaling, with the *tlr5a* morpholino giving a complete block of induction of *il1b* by injection with flagellin and a partial effect of the *tlr5b* morpholino. These data suggests that these two *tlr5* copies function in concert, perhaps by forming heterodimers.

**Fig. 6:**
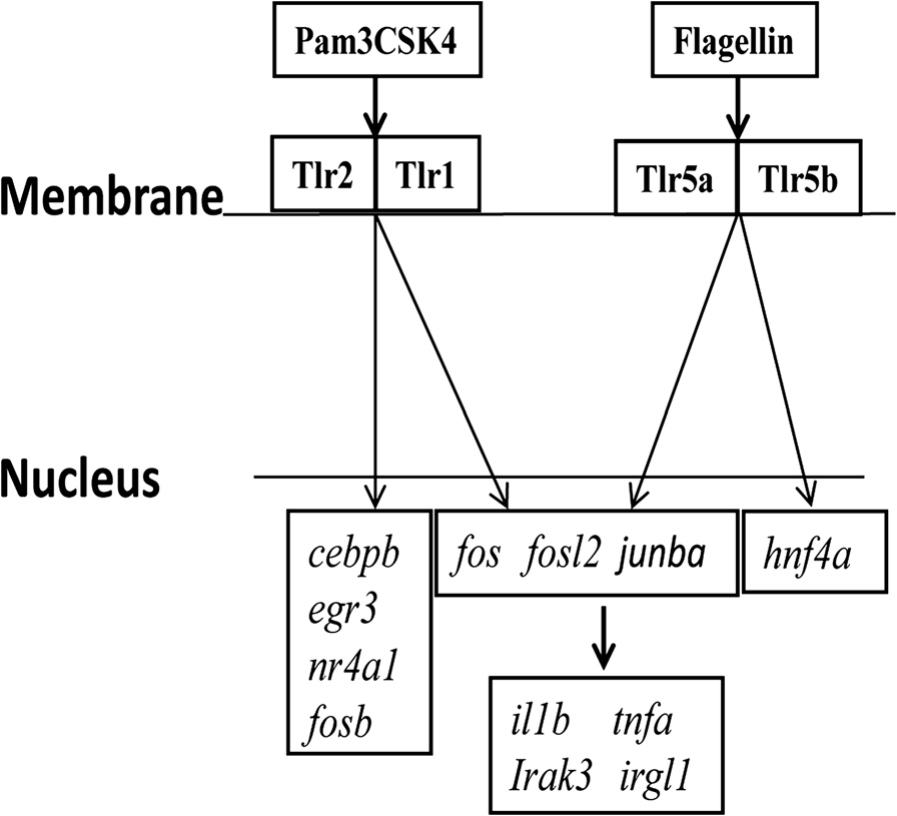
Specific and common responses to Tlr2 and Tlr5 ligands. The whole organism transcriptome response of zebrafish embryos to treatment with the Tlr2 ligand Pam3CSK4 or the Tlr5 ligand flagellin results in the induction of specific and common transcription factor genes as indicated in the figure. The common transcription factor genes (in cooperation with other non-inducible factors, e.g. of the NF-κB family), likely function upstream of the effector genes commonly induced by Pam3CSK4 and flagellin. The transcription factor genes induced by only one of the two ligands are likely to contribute to further specificity in the transcriptional responses of downstream effector genes

An important question that comes from our work is how to explain the difference in gene sets that are regulated in response to Pam3CSK4 and flagellin? (Fig. 6) Since our detection system seems sufficiently sensitive to detect even minor effects on gene transcription, a limitation in dosing is not a likely explanation for this difference, so instead we think of another two possible alternative explanations. In the first place we could speculate that there are specific downstream signaling partners for Tlr2 and Tlr5. However, such partners have not yet been indicated by previous studies, in contrast, there are evidences that all known direct binding partners are common for both Tlr5 and Tlr2 proteins: including the adaptor proteins Myd88, and Tirap (Mal) that have been implicated in signaling of both proteins [52]. Furthermore, the functions of these genes in the direct recognition of TLR2 and TLR5 ligands have not been tested yet in whole animal models. Mutants for Tirap have not been described yet in zebrafish making the specific function of this gene currently difficult to investigate. Another possible explanation is that the differential response of zebrafish embryos to these two PAMPs is the result of an additive effect of the recognition by different cell types. In this case, the common group of activated downstream genes might be encoded by the response of common immune cells which have a full repertoire of Tlr receptors whereas the specific response might be the result of a distinct transcriptional response of specialized cells that do not encode all Tlr receptors. The detailed study of these transcription factors will provide valuable information on the specific immune transcriptional signatures elicited by different pathogens. For this purpose the genetic tractability of the zebrafish system will allow the generation of new reporter lines that will contribute to the understanding of how these responses modulate the innate immune system. In addition, such reporter lines will be of general interest since Pam3CSK4 and flagellin signaling pathways are broadly used to study the Tlrs function in inflammatory microbial infection. Furthermore, this signaling pathway is also relevant for studies of atherosclerosis and autoimmune diseases processes [53, 54]. Therefore the used systemic approach can be highly useful for future studies of a broad spectrum of immune-related diseases.

## Additional files

Additional file 1: Table S1. List of morpholinos and primers. Additional file 2: Table S4. List of mclusters and mappable reads for Pam3CSK4, flagellin and control RNASeq-library. Additional file 3: Figure S1. Change trend of the number of DEGs according to different fold-change and *p-value.*
Additional file 4: Table S5. List of responsive genes to Pam3CSK4 without any selection criteria. Additional file 5: Table S6. List of responsive genes to flagellin without any selection criteria. Additional file 6: Table S3. Fold-change and *p*-value of 80 genes responsive to both Pam3CSK4 and flagellin in WT and *tlr2*- and *tlr5a*-morphants. Additional file 7: Table S2. Fold-change and *p*-value of 48 Pam3CSK4- and 42 flagellin-responsive genes in WT and *tlr* morphants.
