# Supplementary material for: Common and specific downstream signaling targets controlled by Tlr2 and Tlr5 innate immune signaling in zebrafish
Source: BMC Genomics. 2015 Jul 25;16(1):547. doi: 10.1186/s12864-015-1740-9 (PMC4514945; doi:10.1186/s12864-015-1740-9)
Supplement: Additional file 1: Table S1. — List of morpholinos and primers. [file 12864_2015_1740_MOESM1_ESM.docx]

**Supplemental table I**

**List of morpholinos and primers**

| **Name** | **Sequences** |
| --- | --- |
| *tlr2* mo  Sc mo  *ppial*  *il1b*  *tnfa*  *il6*  *il8*  *il10* | 5'- AGTCATTGTTCCTACGAGTCTCATC-3'  5'-CCTCTTACCTCAGTTACAATTTATA-3‘  FW: 5'-ACACTGAAACACGGAGGCAAAG-3‘  RV: 5'-CATCCACAACCTTCCCGAACAC-3‘  FW: 5'-GAACAGAATGAAGCACATCAAACC-3‘  RV: 5'-ACGGCACTGAATCCACCAC-3'  FW: 5'-AGACCTTAGACTGGAGAGATGAC-3'  RV: 5'-CAAAGACACCTGGCTGTAGAC-3‘  FW: 5'-TCAACTTCTCCAGCGTGATG-3'  RV: 5'-TCTTTCCCTCTTTTCCTCCTG-3'  FW: 5'-TGTGTTATTGTTTTCCTGGCATTTC3'  RV: 5'-GCGACAGCGTGGATCTACAG-3‘  FW: 5'-GGAGACCATTCTGCCAACAGC-3'  RV: 5'-TCTTGCATTTCACCATATCCCG-3' |
