# Supplementary material for: Common and specific downstream signaling targets controlled by Tlr2 and Tlr5 innate immune signaling in zebrafish
Source: BMC Genomics. 2015 Jul 25;16(1):547. doi: 10.1186/s12864-015-1740-9 (PMC4514945; doi:10.1186/s12864-015-1740-9)
Supplement: Additional file 2: Table S4. — List of mclusters and mappable reads for Pam3CSK4, flagellin and control RNASeq-library. [file 12864_2015_1740_MOESM2_ESM.docx]

Supplemental table IV

|  |  | Sample1 | Sample2 | Sample3 |
| --- | --- | --- | --- | --- |
| Pam3CSK4 | Mappable reads | 7,991,601 | 7,167,382 | 28,575,686 |
|  | Mclusters | 12,040,507 | 9,959,743 | 38,992,807 |
| Flagellin | Mappable reads | 7,448,933 | 9,718,846 | 7,456,109 |
|  | Mclusters | 11.496.574 | 12,571,938 | 9,552,331 |
| Control | Mappable reads | 12,739,496 | 7,240,312 | 11,715,180 |
|  | Mclusters | 18,893,522 | 9,309,796 | 15,303,450 |
