# Supplementary figures and images for: Common and specific downstream signaling targets controlled by Tlr2 and Tlr5 innate immune signaling in zebrafish
Source: BMC Genomics. 2015 Jul 25;16(1):547. doi: 10.1186/s12864-015-1740-9 (PMC4514945; doi:10.1186/s12864-015-1740-9)

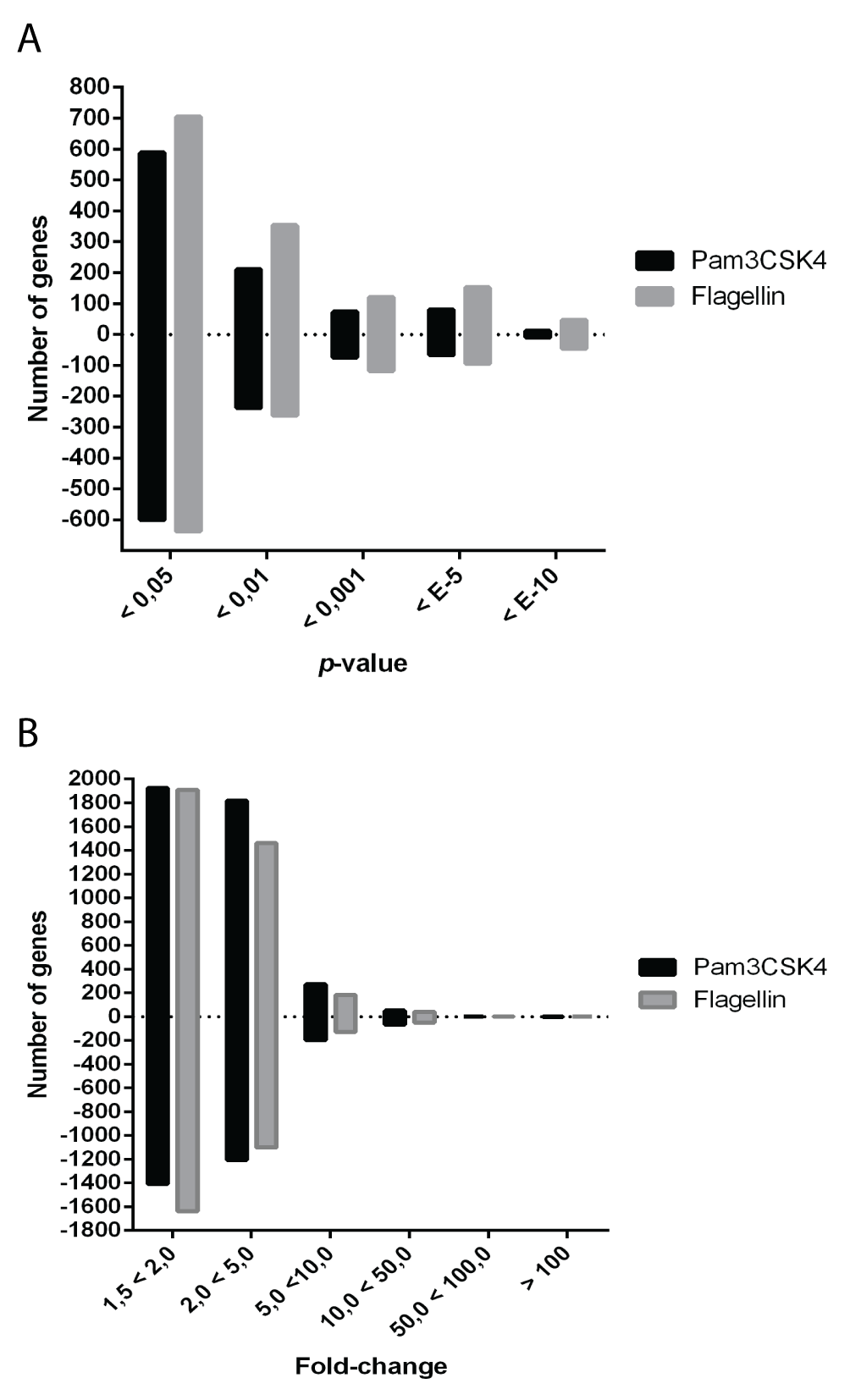

Supplement: Additional file 3: Figure S1. — Change trend of the number of DEGs according to different fold-change and p-value. [file 12864_2015_1740_MOESM3_ESM.tif]
