# Supplementary material for: Common and specific downstream signaling targets controlled by Tlr2 and Tlr5 innate immune signaling in zebrafish
Source: BMC Genomics. 2015 Jul 25;16(1):547. doi: 10.1186/s12864-015-1740-9 (PMC4514945; doi:10.1186/s12864-015-1740-9)
Supplement: Additional file 6: Table S3. — Fold-change and p-value of 80 genes responsive to both Pam3CSK4 and flagellin in WT and tlr2- and tlr5a-morphants. [file 12864_2015_1740_MOESM6_ESM.docx]

**Supplemental table III**

**Fold-change and p-value of 80 common responsive genes in WT and tlr2- and tlr5a- morphants**

|  |  |  | **WT (Pam3CSK4)** | | ***tlr2* MO (Pam3CSK4)** | | **WT (flagellin)** | | ***tlr5a* MO (flagellin)** | |
| --- | --- | --- | --- | --- | --- | --- | --- | --- | --- | --- |
| **Nr.** | **Gene** | **Gene ID** | **Fold-change** | **p-value** | **Fold-change** | ***p*-value** | **Fold-change** | ***p-*value** | **Fold-change** | ***p*-value** |
| 1 | *fabp7b* | ENSDARG00000034650 | 20, 42599844 | 0, 00026115 | 1, 801202013 | 0, 161997114 | 7, 424504962 | 0, 002304713 | 2, 043872979 | 0, 088545403 |
| 2 | *si:dkey-3n22. 9* | ENSDARG00000096273 | 15, 59184373 | 0, 00299433 | 2, 946091276 | 0, 230618229 | 11, 24096053 | 7, 30E-05 | 0, 299146766 | 1 |
| 3 | *tnfb* | ENSDARG00000013598 | 14, 02679213 | 1, 05E-06 | 1, 263912528 | 0, 3910098 | 8, 057948742 | 0, 000578783 | 1, 469706434 | 0, 223771858 |
| 4 | *zgc:162592* | ENSDARG00000060346 | 11, 46916471 | 0, 001166918 | -1, 206025304 | 0, 376172144 | 11, 50351324 | 1, 26E-08 | -1, 171738504 | 0, 341267399 |
| 5 | *C5H11orf1* | ENSDARG00000090173 | 8, 469366169 | 0, 001216631 | 2, 014203875 | 0, 096432202 | 6, 598276848 | 0, 006222754 | 2, 096622722 | 0, 025822605 |
| 6 | *tcap* | ENSDARG00000007344 | 8, 4052304 | 1, 79E-31 | -1, 081197784 | 0, 457323805 | 3, 901720378 | 1, 27E-09 | -1, 960135438 | 0, 006636661 |
| 7 | *si:ch211-51a19. 5* | ENSDARG00000094668 | 8, 021763455 | 0, 004528743 | 1, 605590058 | 0, 36559555 | 4, 884617917 | 0, 008331127 | -1, 199650159 | 0, 370206595 |
| 8 | *fos* | ENSDARG00000031683 | 7, 473649742 | 0, 000132152 | -1, 488185462 | 0, 305926583 | 2, 29535238 | 0, 000248453 | 1, 202788137 | 0, 315444835 |
| 9 | *si:dkey-24c2. 7* | ENSDARG00000092427 | 5, 835322653 | 0, 00878104 | -1, 05098754 | 0, 971532144 | 9, 49455826 | 3, 36E-05 | -2, 06691355 | 0, 121255318 |
| 10 | *si:dkey-242h9. 3* | ENSDARG00000069189 | 5, 403803357 | 4, 02E-05 | -1, 319302108 | 0, 444882355 | 3, 404944785 | 0, 008751826 | 1, 396394836 | 0, 128222388 |
| 11 | *irak3* | ENSDARG00000053131 | 4, 977605877 | 0, 000836463 | -1, 269690102 | 0, 939111075 | 6, 982117758 | 1, 70E-05 | -1, 107394094 | 0, 858564147 |
| 12 | *junba* | ENSDARG00000074378 | 4, 924657234 | 4, 51E-16 | -2, 099904663 | 0, 143756974 | 2, 297792063 | 1, 18E-05 | 1, 29382283 | 0, 122389209 |
| 13 | *si:dkey-13m3. 2* | ENSDARG00000090700 | 4, 794594692 | 3, 39E-05 | 1, 124019957 | 0, 726229743 | 3, 114675333 | 0, 004152052 | 1, 202150058 | 0, 408849083 |
| 14 | *zgc:171490* | ENSDARG00000071643 | 4, 613526402 | 4, 83E-07 | 1, 422887046 | 0, 342829098 | 3, 47122828 | 7, 87E-05 | -1, 27456709 | 0, 218709552 |
| 15 | *arl14* | ENSDARG00000063223 | 4, 516522885 | 4, 59E-09 | -1, 084321987 | 0, 682723738 | 4, 490414904 | 4, 66E-10 | -1, 660005354 | 0, 001112597 |
| 16 | *thnsl2* | ENSDARG00000032584 | 4, 389594488 | 8, 10E-06 | -1, 395261211 | 0, 121877258 | 2, 562259319 | 0, 00779622 | -1, 486934759 | 0, 224493027 |
| 17 | *si:dkey-27h10. 2* | ENSDARG00000094485 | 4, 319268659 | 0, 002914043 | 1, 540726452 | 0, 272902643 | 3, 262804265 | 0, 005038336 | -1, 084040223 | 0, 760684934 |
| 18 | *drd3* | ENSDARG00000032131 | 4, 242544688 | 0, 002604575 | 1, 047785324 | 1 | 2, 153224541 | 0, 008470068 | 1, 16983498 | 0, 386487177 |
| 19 | *il1b* | ENSDARG00000005419 | 3, 847495455 | 1, 39E-06 | -1, 170164654 | 0, 740093623 | 3, 229488052 | 1, 51E-05 | -1, 120945699 | 0, 482835861 |
| 20 | *si:ch211-279m15. 1* | ENSDARG00000092900 | 3, 697158803 | 6, 73E-06 | 1, 278170091 | 0, 255136891 | 2, 318288729 | 0, 005473253 | 1, 219353097 | 0, 25514997 |
| 21 | *zgc:153921* | ENSDARG00000020738 | 3, 653884272 | 5, 70E-05 | 1, 896555422 | 0, 053270505 | 4, 400174205 | 1, 25E-07 | 1, 347884773 | 0, 331946879 |
| 22 | *hspb9* | ENSDARG00000078674 | 3, 615545879 | 4, 21E-23 | 1, 103097796 | 0, 465011032 | 3, 718308911 | 2, 68E-43 | -1, 600694007 | 0, 005596722 |
| 23 | *card9* | ENSDARG00000067672 | 3, 596214162 | 0, 001574838 | 1, 28775398 | 0, 358360673 | 2, 914195207 | 0, 001766592 | 1, 507626651 | 0, 047008275 |
| 24 | *CU929219. 1* | ENSDARG00000086739 | 3, 418978686 | 0, 001683955 | -1, 20186176 | 0, 695331788 | 3, 017839574 | 0, 001655579 | 1, 030963904 | 0, 871119839 |
| 25 | *tmem8c* | ENSDARG00000026001 | 3, 332483469 | 0, 000166145 | 2, 15730978 | 0, 08063024 | 3, 580199427 | 1, 05E-05 | 1, 319863842 | 0, 114000888 |
| 26 | *gpr84* | ENSDARG00000077308 | 3, 295535353 | 1, 39E-06 | -1, 343394175 | 0, 229510692 | 3, 037972964 | 5, 28E-06 | 1, 235769207 | 0, 208384106 |
| 27 | *STYK1 (2 of 3)* | ENSDARG00000070950 | 3, 190539203 | 0, 007577865 | -1, 000685602 | 0, 880124388 | 3, 109062727 | 0, 004566149 | 1, 183805713 | 0, 405812446 |
| 28 | *si:ch211-251f6. 6* | ENSDARG00000053448 | 3, 015412341 | 0, 002644296 | -1, 041897936 | 0, 83823624 | 6, 032053714 | 2, 18E-31 | -1, 458421384 | 0, 115884942 |
| 29 | *fosl2* | ENSDARG00000040623 | 2, 863034694 | 5, 94E-08 | -1, 53037668 | 0, 134734136 | 2, 198773482 | 5, 79E-11 | -1, 084221284 | 0, 494988805 |
| 30 | *si:dkey-169l5. 3* | ENSDARG00000015530 | 2, 852861461 | 0, 00010616 | 1, 658356344 | 0, 14903095 | 2, 18377551 | 0, 006143272 | 1, 275970141 | 0, 166329083 |
| 31 | *hspb11* | ENSDARG00000002204 | 2, 813237597 | 3, 42E-10 | 1, 491655632 | 0, 017997047 | 3, 33631667 | 1, 40E-20 | -1, 34745076 | 0, 096460625 |
| 32 | *cyp24a1* | ENSDARG00000070420 | 2, 787622724 | 0, 00018713 | -1, 29931851 | 0, 34989433 | 2, 135712466 | 0, 001752119 | -1, 124424857 | 0, 344470181 |
| 33 | *si:dkey-51d8. 9* | ENSDARG00000096217 | 2, 765356963 | 0, 009958644 | 1, 055313088 | 0, 981819507 | 3, 160165018 | 0, 000649349 | -1, 402656117 | 0, 152401759 |
| 34 | *wu:fj08f03* | ENSDARG00000077169 | 2, 691260015 | 0, 000163352 | -1, 225055068 | 0, 35614244 | 2, 034158486 | 1, 76E-06 | 1, 08253159 | 0, 545242521 |
| 35 | *b3gnt5b* | ENSDARG00000004396 | 2, 670763778 | 0, 000522073 | 1, 155347317 | 0, 362582272 | 2, 677581447 | 0, 003051091 | 1, 144792787 | 0, 439206707 |
| 36 | *CU638740. 1* | ENSDARG00000074971 | 2, 56572927 | 0, 000847471 | 1, 25300215 | 0, 480732572 | 3, 230080663 | 8, 91E-07 | 1, 19593287 | 0, 504835408 |
| 37 | *CU302436. 1* | ENSDARG00000018566 | 2, 558938796 | 2, 32E-05 | 1, 117551984 | 0, 599197843 | 2, 981550266 | 9, 54E-19 | -1, 756897468 | 3, 62E-05 |
| 38 | *si:dkey-103d23. 3* | ENSDARG00000095057 | 2, 527947651 | 0, 005528325 | 1, 936577793 | 0, 026346477 | 2, 478678119 | 0, 004189131 | -1, 102369046 | 0, 589296241 |
| 39 | *BX000701. 3* | ENSDARG00000094216 | 2, 501675497 | 0, 002983715 | 2, 268791036 | 0, 071516155 | 3, 013564842 | 3, 97E-05 | 1, 315090936 | 0, 149270562 |
| 40 | *ddb2* | ENSDARG00000041140 | 2, 449341304 | 3, 13E-06 | 1, 014290421 | 0, 84089841 | 2, 290792008 | 3, 86E-07 | 1, 589577576 | 0, 001541958 |
| 41 | *abraa* | ENSDARG00000038583 | 2, 388377779 | 8, 78E-08 | 1, 069315216 | 0, 883265398 | 2, 679089041 | 4, 25E-14 | -1, 69335118 | 0, 001096718 |
| 42 | *ncf1* | ENSDARG00000033735 | 2, 261718965 | 5, 91E-05 | 1, 627923882 | 0, 050695409 | 2, 210452931 | 9, 32E-06 | 1, 508040722 | 0, 01158271 |
| 43 | *irg1l* | ENSDARG00000062788 | 2, 241732878 | 0, 000749161 | -1, 005057856 | 0, 920679646 | 3, 454001817 | 6, 74E-34 | 1, 407007279 | 0, 005630154 |
| 44 | *xirp1* | ENSDARG00000030722 | 2, 203534134 | 1, 42E-12 | 1, 385329246 | 0, 009725561 | 2, 713645659 | 4, 07E-33 | -1, 325882243 | 0, 020390503 |
| 45 | *noxo1a* | ENSDARG00000041294 | 2, 199393719 | 4, 72E-07 | -1, 128460766 | 0, 608634 | 2, 355334789 | 2, 45E-10 | 1, 529807186 | 0, 001130733 |
| 46 | *si:dkey-54i3. 3_2* | ENSDARG00000093652 | 2, 185084278 | 2, 12E-05 | -1, 247456152 | 0, 19876595 | 2, 866160391 | 7, 73E-11 | -1, 107566312 | 0, 449126101 |
| 47 | *sb:cb252* | ENSDARG00000058206 | 2, 112654772 | 0, 006711631 | 1, 306276114 | 0, 127618665 | 2, 929247966 | 6, 97E-27 | 1, 072414773 | 0, 75431129 |
| 48 | *tefa* | ENSDARG00000039117 | 2, 076745319 | 3, 91E-05 | -1, 013097815 | 0, 950219272 | 2, 310499666 | 5, 98E-24 | 1, 46145106 | 0, 00227304 |
| 49 | *CU914813. 1* | ENSDARG00000088885 | -2, 032372984 | 1, 04E-10 | 1, 513752735 | 0, 000457555 | -2, 215667122 | 1, 39E-16 | 1, 386945808 | 0, 330011417 |
| 50 | *galk2* | ENSDARG00000004059 | -2, 035838739 | 0, 000442982 | 1, 119816441 | 0, 702125677 | -9, 324149695 | 1, 15E-17 | 1, 338547711 | 0, 088800739 |
| 51 | *crygm2d21* | ENSDARG00000086658 | -2, 127003088 | 0, 000771535 | -2, 522197791 | 0, 077350885 | -5, 271158992 | 1, 02E-10 | -2, 379280512 | 0, 035776391 |
| 52 | *mybpc1* | ENSDARG00000045560 | -2, 179466998 | 0, 003972577 | -2, 02823292 | 0, 248697108 | -13, 88549107 | 2, 92E-13 | 1, 253653082 | 0, 228665588 |
| 53 | *si:ch211-209l18. 4* | ENSDARG00000090847 | -2, 326277955 | 0, 000787692 | 1, 413817866 | 0, 390656393 | -2, 252248226 | 0, 000463858 | 1, 428382332 | 0, 113013985 |
| 54 | *gpnmb* | ENSDARG00000062688 | -2, 340269415 | 3, 34E-06 | -1, 425854837 | 0, 114201454 | -2, 903849407 | 4, 28E-13 | -1, 362069926 | 0, 097479392 |
| 55 | *zgc:195001* | ENSDARGf00000070484 | -2, 343452924 | 8, 39E-06 | -1, 331651726 | 0, 328288242 | -2, 420471275 | 1, 79E-09 | -1, 639147233 | 0, 004321891 |
| 56 | *crygm2d2* | ENSDARG00000086917 | -2, 367678102 | 0, 000150063 | -1, 980903794 | 0, 117027487 | -4, 587323362 | 6, 16E-10 | -3, 110642193 | 0, 03579882 |
| 57 | *obscn* | ENSDARG00000043309 | -2, 420008419 | 0, 0084174 | -1, 780218262 | 0, 059809356 | -3, 031248963 | 3, 63E-05 | -1, 432617231 | 0, 046463636 |
| 58 | *si:dkeyp-113d7. 4* | ENSDARG00000090268 | -2, 423608445 | 1, 63E-19 | -1, 300894766 | 0, 1507735 | -2, 94829839 | 2, 67E-62 | -1, 291731675 | 0, 139511837 |
| 59 | *dnah9* | ENSDARG00000004221 | -2, 652731201 | 8, 82E-05 | 1, 05514548 | 0, 960134992 | -2, 255162079 | 0, 000861961 | -1, 216237129 | 0, 758923512 |
| 60 | *si:dkey-113d16. 9* | ENSDARG00000096776 | -2, 713359688 | 0, 0080841 | 1, 188029605 | 0, 546859521 | -2, 538716417 | 0, 007044934 | 1, 015022575 | 0, 980623478 |
| 61 | *caspb* | ENSDARG00000052039 | -2, 899441532 | 8, 33E-05 | 1, 257863691 | 0, 372300412 | -4, 705992611 | 1, 15E-11 | -1, 623572001 | 0, 09802334 |
| 62 | *aglb* | ENSDARG00000016491 | -3, 190654125 | 0, 001016368 | 1, 016310121 | 0, 964688692 | -2, 459243591 | 0, 002765452 | -2, 094853289 | 0, 076585198 |
| 63 | *BX296557. 6* | ENSDARG00000090280 | -3, 249883098 | 1, 16E-05 | -1, 065076716 | 0, 781621722 | -2, 155528622 | 0, 006686404 | 2, 322227499 | 0, 081491013 |
| 64 | *zgc:158463* | ENSDARG00000089382 | -3, 271343666 | 7, 74E-08 | -1, 118463938 | 0, 919394308 | -2, 200503269 | 0, 001688937 | 2, 274428217 | 0, 137679721 |
| 65 | *BX296557. 3* | ENSDARG00000086686 | -3, 273230267 | 0, 001633722 | 1, 053376188 | 0, 559481436 | -2, 648190319 | 0, 006920869 | 2, 071686554 | 0, 165363972 |
| 66 | *BX296557. 4* | ENSDARG00000088313 | -3, 281608789 | 3, 11E-05 | -1, 009714904 | 0, 656776059 | -2, 246995467 | 0, 003084828 | 2, 225354348 | 0, 094789174 |
| 67 | *BX537263. 2* | ENSDARG00000087337 | -3, 366637768 | 1, 74E-06 | 1, 07690224 | 0, 585042048 | -2, 218721227 | 0, 004110984 | 2, 134557344 | 0, 071766739 |
| 68 | *si:dkey-153m14. 1* | ENSDARG00000096403 | -3, 492434322 | 6, 10E-12 | -1, 17609782 | 1 | -2, 296737251 | 8, 66E-06 | 2, 38083607 | 0, 063394214 |
| 69 | *tmem117* | ENSDARG00000088356 | -3, 511280749 | 0, 000766159 | -1, 309832152 | 0, 394706152 | -2, 138971474 | 0, 007918725 | -1, 473596223 | 0, 073739071 |
| 70 | *si:dkey-111b14. 2* | ENSDARG00000096145 | -3, 811480818 | 0, 000223362 | 1, 01219326 | 0, 658211163 | -2, 41249831 | 0, 002319574 | 3, 118184689 | 0, 08167801 |
| 71 | *CT956064. 3* | ENSDARG00000088436 | -3, 851729885 | 6, 82E-06 | -1, 289476415 | 0, 835512664 | -2, 414782514 | 0, 001991469 | 2, 212415778 | 0, 078210652 |
| 72 | *BX296557. 7* | ENSDARG00000091744 | -3, 921974806 | 1, 12E-07 | -1, 146314308 | 1 | -2, 227839415 | 0, 001773358 | 2, 192565357 | 0, 103082348 |
| 73 | *AL935186. 4* | ENSDARG00000085168 | -4, 24792884 | 2, 53E-06 | -1, 754990884 | 0, 305338279 | -2, 179087934 | 0, 005366196 | 2, 412917082 | 0, 025095297 |
| 74 | *CT583728. 2* | ENSDARG00000086372 | -4, 264070558 | 1, 55E-06 | -2, 034091236 | 0, 166390466 | -2, 019262799 | 0, 00160466 | 2, 42484504 | 0, 020721458 |
| 75 | *CT583728. 11* | ENSDARG00000088311 | -4, 304853064 | 0, 001260354 | -1, 016242626 | 0, 819022964 | -2, 7616031 | 0, 009831456 | 2, 335945247 | 0, 127023464 |
| 76 | *CT956064. 4* | ENSDARG00000088494 | -4, 741587744 | 1, 35E-05 | 1, 045690502 | 0, 620438412 | -2, 003886726 | 0, 009033269 | 2, 437493213 | 0, 098416573 |
| 77 | *ponzr3* | ENSDARG00000088589 | -5, 135925898 | 0, 00208668 | -2, 867222459 | 0, 184455737 | -3, 527190795 | 0, 005511146 | -1, 114557443 | 0, 991188213 |
| 78 | *BX537263. 12* | ENSDARG00000090733 | -6, 30665423 | 7, 64E-06 | -1, 059730842 | 0, 725353605 | -2, 369398107 | 0, 004883946 | 2, 234989801 | 0, 056074143 |
| 79 | *lim2. 5* | ENSDARG00000041295 | -6, 569232177 | 0, 002833848 | -4, 066761793 | 0, 094160338 | -3, 775122784 | 0, 005333233 | -1, 264262941 | 0, 570718879 |
| 80 | *CT956064. 2* | ENSDARG00000087315 | -11, 14706649 | 2, 18E-06 | -2, 341489955 | 0, 150066515 | -2, 729560786 | 0, 003574594 | 2, 207080313 | 0, 029868523 |
