# Supplementary material for: Common and specific downstream signaling targets controlled by Tlr2 and Tlr5 innate immune signaling in zebrafish
Source: BMC Genomics. 2015 Jul 25;16(1):547. doi: 10.1186/s12864-015-1740-9 (PMC4514945; doi:10.1186/s12864-015-1740-9)
Supplement: Additional file 7: Table S2. — Fold-change and p-value of 48 Pam3CSK4- and 42 flagellin-responsive genes in WT and tlr morphants. [file 12864_2015_1740_MOESM7_ESM.docx]

**Supplemental table II**

**Fold-change and *p*-value of 48 Pam3CSK4- and 42 flagellin-responsive genes in WT and *tlr* morphants**

|  |  | **Wild type** | | ***tlr2* morphants** | |
| --- | --- | --- | --- | --- | --- |
| **Gene** | **Gene ID** | **Fold-change** | ***p*-value** | **Fold-change** | ***p*-value** |
| *si:dkey-287i17. 3* | ENSDARG00000093647 | 37, 13822 | 0, 003475 | -1, 11067 | 1 |
| *egr3* | ENSDARG00000089156 | 7, 592804 | 1, 34E-08 | -4, 36467 | 0, 004329 |
| *upk3l* | ENSDARG00000092872 | 5, 507635 | 0, 005188 | -1, 50148 | 0, 552857 |
| *si:dkeyp-116h7. 2* | ENSDARG00000092632 | 4, 667721 | 1, 24E-06 | -1, 12901 | 0, 847042 |
| *fosl1a* | ENSDARG00000015355 | 4, 002537 | 3, 00E-16 | -3, 36469 | 0, 003299 |
| *fosb* | ENSDARG00000055751 | 3, 98386 | 0, 000438 | -2, 07748 | 0, 001886 |
| *gda* | ENSDARG00000002986 | 3, 956519 | 0, 000702 | -1, 25968 | 0, 64589 |
| *CU019646. 2* | ENSDARG00000091234 | 3, 923755 | 7, 97E-06 | -2, 08687 | 0, 081899 |
| *kcnj1a. 6* | ENSDARG00000088484 | 3, 478039 | 2, 67E-06 | -1, 38649 | 0, 206858 |
| *CU367845. 1* | ENSDARG00000071384 | 3, 375473 | 0, 004943 | 1, 135737 | 0, 625199 |
| *IER2* | ENSDARG00000086881 | 3, 290588 | 3, 17E-07 | 1, 169767 | 0, 513637 |
| *si:dkey-15h8. 11* | ENSDARG00000075474 | 3, 237288 | 0, 008588 | -1, 01265 | 1 |
| *zgc:152891* | ENSDARG00000016789 | 3, 151323 | 0, 006665 | 1, 232991 | 0, 922887 |
| *atp5ia* | ENSDARG00000078113 | 3, 022055 | 1, 06E-08 | 1, 668462 | 0, 006593 |
| *plek2* | ENSDARG00000012789 | 2, 994366 | 0, 000113 | -2, 01262 | 0, 152512 |
| *pnp5a* | ENSDARG00000078619 | 2, 930795 | 9, 62E-08 | -1, 39776 | 0, 051383 |
| *kcnj1a. 3* | ENSDARG00000086248 | 2, 878537 | 0, 002284 | -1, 49152 | 0, 42657 |
| *elovl7b* | ENSDARG00000005785 | 2, 865127 | 8, 83E-06 | -1, 35179 | 0, 035254 |
| *BX005256. 1* | ENSDARG00000040284 | 2, 606485 | 0, 000705 | -1, 35851 | 0, 403852 |
| *CABZ01072534. 1* | ENSDARG00000075951 | 2, 482739 | 5, 32E-05 | -1, 79229 | 0, 63581 |
| *dok1a* | ENSDARG00000078108 | 2, 481505 | 0, 009457 | -1, 00574 | 0, 928145 |
| *stard10* | ENSDARG00000031929 | 2, 473433 | 1, 63E-07 | -1, 22535 | 0, 315112 |
| *nr4a1* | ENSDARG00000000796 | 2, 397662 | 0, 00182 | -1, 2438 | 0, 158493 |
| *tuft1a* | ENSDARG00000061242 | 2, 340398 | 0, 000686 | -2, 10162 | 0, 002522 |
| *CHST5* | ENSDARG00000061357 | 2, 29841 | 0, 000974 | -1, 15192 | 0, 632852 |
| *ZNF135 (4 of 10)* | ENSDARG00000088352 | 2, 248742 | 0, 008201 | -1, 61308 | 0, 308616 |
| *ptgis* | ENSDARG00000060094 | 2, 229887 | 0, 002754 | -1, 4492 | 0, 21328 |
| *cebpb* | ENSDARG00000042725 | 2, 158254 | 5, 65E-11 | -2, 10519 | 0, 029399 |
| *SIK1* | ENSDARG00000058606 | 2, 144074 | 5, 96E-10 | -1, 66384 | 0, 050379 |
| *ovol1* | ENSDARG00000076472 | 2, 135477 | 0, 003806 | 1, 195271 | 0, 270324 |
| *dusp4* | ENSDARG00000044688 | 2, 111464 | 3, 85E-05 | -1, 16768 | 0, 279795 |
| *ccrl1b* | ENSDARG00000040133 | 2, 094717 | 0, 003065 | -1, 7224 | 0, 159897 |
| *dmbx1b* | ENSDARG00000002510 | 2, 077321 | 0, 008432 | -1, 49531 | 0, 377086 |
| *THRAP3 (3 of 3)* | ENSDARG00000044459 | 2, 063407 | 0, 000563 | -1, 09017 | 0, 648443 |
| *ptprjb* | ENSDARG00000091539 | 2, 063372 | 0, 004662 | 1, 203162 | 0, 4403 |
| *si:ch73-27e22. 6* | ENSDARG00000031731 | 2, 051445 | 0, 002219 | 1, 176148 | 0, 693489 |
| *pfdn6* | ENSDARG00000037108 | 2, 043484 | 0, 000432 | 1, 492501 | 0, 006108 |
| *junbb* | ENSDARG00000088371 | 2, 034555 | 5, 83E-07 | -1, 83174 | 0, 079101 |
| *adam28* | ENSDARG00000035514 | 2, 026869 | 0, 000287 | -1, 40736 | 0, 132695 |
| *zgc:64022* | ENSDARG00000070705 | 2, 020163 | 0, 009515 | 1, 103561 | 0, 543534 |
| *BX649384. 1* | ENSDARG00000035657 | -2, 03065 | 0, 002598 | -1, 26291 | 0, 530954 |
| *MYO1G* | ENSDARG00000036104 | -2, 08606 | 0, 007181 | 1, 174045 | 0, 650351 |
| *ghitm* | ENSDARG00000014573 | -2, 17879 | 0, 003117 | -1, 97913 | 0, 065987 |
| *GBF1 (2 of 2)* | ENSDARG00000088406 | -2, 21319 | 0, 002901 | 1, 114998 | 0, 99387 |
| *wu:fc75a09* | ENSDARG00000089342 | -2, 24283 | 0, 002886 | 1, 407139 | 0, 22163 |
| *gpr123* | ENSDARG00000054177 | -2, 45787 | 0, 000664 | 1, 403487 | 0, 269881 |
| *CRP* | ENSDARG00000045089 | -2, 7389 | 9, 84E-05 | -1, 75203 | 0, 115213 |
| *BX548011. 4* | ENSDARG00000088938 | -3, 71814 | 0, 003472 | -1, 30874 | 0, 967643 |
|  |  | **Wild type** | | ***tlr5a* morphants** | |
| **Gene** | **Gene ID** | **Fold-change** | **p-value** | **Fold-change** | **p-value** |
| *si:ch211-222e20. 4* | ENSDARG00000092578 | -2, 06639 | 0, 002116 | 8, 78476 | 8, 43E-13 |
| *csrp3* | ENSDARG00000069975 | 4, 365181 | 0, 001141 | -3, 44199 | 0, 004287 |
| *arsh* | ENSDARG00000002299 | 2, 001475 | 0, 000857 | -1, 13328 | 0, 794013 |
| *hnf4a* | ENSDARG00000021494 | 2, 177905 | 1, 66E-07 | -1, 17787 | 0, 267508 |
| *hsp70. 3* | ENSDARG00000021924 | 4, 406201 | 7, 37E-45 | -1, 88916 | 0, 042675 |
| *mcm5* | ENSDARG00000029688 | 4, 753914 | 6, 77E-39 | -1, 72904 | 0, 07748 |
| *zgc:154142* | ENSDARG00000030893 | 2, 136625 | 0, 007184 | 1, 106438 | 0, 687613 |
| *dnajb1b* | ENSDARG00000041394 | 2, 033462 | 3, 96E-10 | 1, 142003 | 0, 412278 |
| *hsp70l* | ENSDARG00000055723 | 6, 425481 | 1, 32E-14 | -1, 64703 | 0, 064216 |
| *adam8b* | ENSDARG00000057644 | 2, 18703 | 3, 15E-05 | -1, 06114 | 0, 760436 |
| *si:ch211-132b12. 7* | ENSDARG00000068374 | 2, 463502 | 0, 002158 | -1, 24973 | 0, 245046 |
| *si:ch211-236g6. 1* | ENSDARG00000071590 | 2, 654147 | 0, 001083 | 1, 854154 | 0, 027412 |
| *si:ch73-341p18. 7* | ENSDARG00000075855 | 2, 213646 | 1, 21E-10 | 1, 153595 | 0, 482278 |
| *fam155a* | ENSDARG00000075858 | 2, 329426 | 0, 000618 | -1, 10943 | 0, 887605 |
| *PRKD1* | ENSDARG00000075949 | 2, 098108 | 0, 003115 | 1, 299394 | 0, 271224 |
| *F7 (3 of 3)* | ENSDARG00000076045 | 2, 227015 | 0, 00959 | 1, 31113 | 0, 217941 |
| *f10* | ENSDARG00000088581 | 2, 18269 | 1, 84E-11 | 1, 015987 | 0, 881408 |
| *CABZ01117452. 1* | ENSDARG00000089416 | 3, 155001 | 0, 002364 | 1, 478251 | 0, 243351 |
| *BX001033. 2* | ENSDARG00000090375 | 2, 001831 | 1, 47E-07 | -1, 1727 | 0, 312002 |
| *si:ch211-213a13. 1* | ENSDARG00000090600 | 2, 193572 | 0, 006997 | -1, 27074 | 0, 324503 |
| *CU638714. 1* | ENSDARG00000091108 | 2, 283161 | 1, 12E-13 | 1, 312361 | 0, 031611 |
| *si:dkey-121n8. 8* | ENSDARG00000092025 | 2, 686128 | 0, 005378 | 1, 19746 | 0, 573243 |
| *hsp70. 2* | ENSDARG00000092362 | 4, 695923 | 5, 69E-26 | -1, 8346 | 0, 070069 |
| *si:dkey-286j17. 4* | ENSDARG00000093402 | 2, 202756 | 0, 000441 | 1, 377617 | 0, 221986 |
| *si:ch211-198k9. 11* | ENSDARG00000096643 | 7, 873719 | 0, 001373 | 1, 616032 | 0, 443109 |
| *six6a* | ENSDARG00000025187 | -2, 61294 | 0, 00413 | 1, 348038 | 0, 20036 |
| *foxg1b* | ENSDARG00000032705 | -2, 65489 | 0, 000274 | -1, 17776 | 0, 456827 |
| *capn2l* | ENSDARG00000034211 | -2, 07178 | 6, 79E-12 | -1, 13901 | 0, 286025 |
| *cryba2b* | ENSDARG00000041925 | -2, 5141 | 5, 17E-09 | -1, 51843 | 0, 008732 |
| *si:dkey-4e7. 3* | ENSDARG00000042124 | -2, 32991 | 9, 32E-05 | 1, 081001 | 0, 743961 |
| *cpt1b* | ENSDARG00000058285 | -2, 32965 | 0, 004626 | -1, 32204 | 0, 149046 |
| *col17a1a* | ENSDARG00000069415 | -2, 06033 | 0, 000223 | -1, 11303 | 0, 629187 |
| *MUC5AC (2 of 3)* | ENSDARG00000070331 | -2, 32786 | 1, 83E-14 | -1, 06961 | 0, 84691 |
| *uroc1* | ENSDARG00000070394 | -2, 89642 | 4, 29E-08 | -1, 04304 | 0, 831642 |
| *agr2* | ENSDARG00000070480 | -2, 29278 | 1, 07E-07 | 1, 126283 | 0, 584978 |
| *sparcl1* | ENSDARG00000074989 | -2, 36568 | 0, 003589 | -1, 76119 | 0, 040298 |
| *CU468012. 1* | ENSDARG00000075205 | -2, 00689 | 0, 007334 | -1, 02333 | 1 |
| *CR388079. 2* | ENSDARG00000088623 | -7, 19881 | 0, 006399 | -1, 04833 | 0, 902528 |
| *Crygm2d17* | ENSDARG00000088687 | -2, 70443 | 1, 83E-05 | -2, 42871 | 0, 020704 |
| *si:ch1073-382c16. 2* | ENSDARG00000092665 | -2, 34498 | 0, 003899 | -1, 89109 | 0, 061621 |
| *acbd7* | ENSDARG00000094730 | -2, 04446 | 0, 003682 | -1, 00161 | 0, 98751 |
| *si:dkey-247i3. 7* | ENSDARG00000095997 | -2, 36786 | 8, 50E-06 | -1, 11526 | 0, 597666 |
